# A novel non-invasive efficient photography-based technique for length measuring and individual identification of seahorses

**DOI:** 10.1038/s41598-023-45420-9

**Published:** 2023-10-21

**Authors:** Diego Luzzatto, Victor Cussac

**Affiliations:** 1 Instituto Andino Patagónico de Tecnologías Biológicas y Geoambientales (IPATEC), Universidad Nacional del Comahue (UNCo) – Consejo Nacional de Investigaciones Científicas y Técnicas (CONICET), R8400 Bariloche, Argentina; 2 Seahorse, Pipefish and Seadragon Specialist Group, IUCN Species Survival Commission, Rue Mauverney 28, 1196 Gland, Switzerland

**Keywords:** Biological techniques, Ecology, Zoology

## Abstract

This study aimed to develop a non-invasive and efficient method for measuring and identifying individual seahorses (*Hippocampus patagonicus*) in their natural habitat. A total of 976 seahorses were captured and photographed on a measuring board to obtain standard length (*L*_s_) measurements. Head photographs were also taken for individual recognition, and a set of 100 seahorses were tagged with visible implant elastomers (VIE) to verify the correspondence between photograph recognition and the applied tags. The analysis showed no significant difference between left and right *L*_s_ measurements. The unique pattern of white dots on the heads served as individual fingerprints, consistent with VIE tagging. The recapture rate was 12%, with 89 individuals observed multiple times. Two distinct growth patterns were identified: males exhibited higher growth rates and a negative correlation with *L*_s_ compared to females. Released seahorses exhibited significantly different behaviors that were related to their sizes (*L*_s_). Smaller seahorses tended to swim slowly towards nearby holdfasts, while larger seahorses escaped further or remained rigid before grasping a holdfast. The proposed methodology allowed for estimating individual seahorse growth rates, and the measurements were objective and precise. The results were obtained through quick and minimally invasive manipulation of the observed individuals.

## Introduction

Ensuring the survival and minimal disturbance of wild fish following a research survey event is an uncommon occurrence. Nonetheless, some nonlethal and minimally invasive techniques have been developed^1^. Often, certain measurements require the removal of animals from their natural habitat. This introduces uncertainty regarding the potential disturbance caused to the population^2^. Assessing fish abundance and size distribution, crucial for fishery management and conservation status estimation, often necessitates removing individuals from their habitat. However, this approach can compromise the accuracy and trend analysis of population parameter estimations, particularly where the fish are rare^3^. Furthermore, populations facing conservation issues are frequently ethically or legally protected from disturbances. This underscores the need for nonlethal and noninvasive methods to collect life history data^4^.

Seahorses (genus *Hippocampus*) are an iconic example of fish species found at low densities and patchily distributed on the sea bottom. Many seahorse species are locally and/or internationally classified as being at risk of extinction, raising concerns for their conservation. The International Union for Conservation of Nature (IUCN) lists 42 species, with two classified as Endangered (EN), 12 as Vulnerable (VU), one as Near Threatened (NT), 10 as Least Concern (LC), and the remaining 17 as Data Deficient (DD). While there is a growing trend in recent ecological studies to avoid collecting wild seahorses or causing disturbances to their habitat, this approach can limit the acquisition of valuable parameters (e.g., accurate length measurements) necessary for their conservation and management^5^.

Measuring seahorses presents unique challenges. Their head and trunk form an approximately right angle, and they have articulated neck and tail junctions, along with their curved trunks. Using a single straight line (i.e., height) for measurements of live seahorses poses constraints for obtaining objective, accurate, and rapid measurements. Most measurement options employed in the field, whether direct by height or indirect (by the sum of various measurements)^6^, introduce considerable bias in assessing seahorse length. Remarkably, the caudal fin is absent in seahorses, so *L*_s_, the single line between the tip of the mouth/snout and the base of the caudal fin, is equal to *L*_t_^7^.

Several methods have been developed for the capture and recapture of seahorses. The most commonly used technique in recent studies is the application of visible implant elastomers (VIE) tags. This technique involves injecting seahorses with colorants, some of which are fluorescent and long-lasting. Given seahorses' body structure, characterized by segmented bony plates and spines, these marks can be uniquely applied, allowing for individual recognition upon recapture^8,9^. In addition to VIE tags, other studies have employed individual recognition methods based on the pattern of bright white dots found on the seahorses' head and body, as well as the contour and shape of the coronet^10–12^.

Conducting population studies on seahorses also poses challenges due to the limited time and high costs associated with underwater sampling, particularly in low density seahorse areas^13^. The constraints of decompression safety and limited air supply determine the available time for data acquisition. Given these time limitations, minimizing the time spent on each measurement becomes desirable during field surveys, allowing more time for searching the next individual. A strategy that involves measuring, recording sex, pregnancy status, color, etc., and applying a VIE tag before releasing the sampled animal could significantly reduce search time and potentially result in a lower number of individuals sampled per survey.

Photography has proven to be a valuable tool in scientific research, allowing for the capture and documentation of various natural phenomena to facilitate subsequent analysis^14^. In this study, we propose and test a new, non-invasive, and effective protocol for obtaining measurements and individual identification of the Patagonian seahorse, *Hippocampus patagonicus* (Piacentino & Luzzatto, 2004), using underwater photography.

## Materials and methods

### Species description

*Hippocampus patagonicus*, a threatened species classified as Vulnerable (VU) by the International Union for Conservation of Nature (IUCN)^15^, faces numerous threats including habitat degradation, coastal development, destructive fishing practices, targeted exploitation, and incidental capture in shrimp trawl fisheries. This species is found in the coastal areas of the Southwestern Atlantic Ocean, ranging from Rio de Janeiro (22° S, Brazil) to Puerto Madryn (42° 47′ S, Argentina). It primarily occupies restricted areas at depths below 20 m, although isolated records indicate occurrences in deeper locations^16,17^. *Hippocampus patagonicus* exhibits habitat associations with various substrates, including marine algae, floating Sargassum species, seagrasses, artificial structures, and sessile invertebrates such as sponges, ascidians, and polychaete worms^18,19^.

### Sampling procedure

The samplings were conducted in a single area of the southernmost known population of the Patagonian seahorse, located in San Antonio Bay in the Rio Negro Province of Argentina, adjacent to a beach resort named Punta Verde (40° 43′ 44″ S 64° 54′ 46″ W), between January 2018 and December 2022. Sampling events were conducted at this area, commencing prior to the predicted lowest tide height for the day. These events were carried out using snorkeling in a shallow channel with depths barely reaching 1 m during low tides.

**Figure 1:**
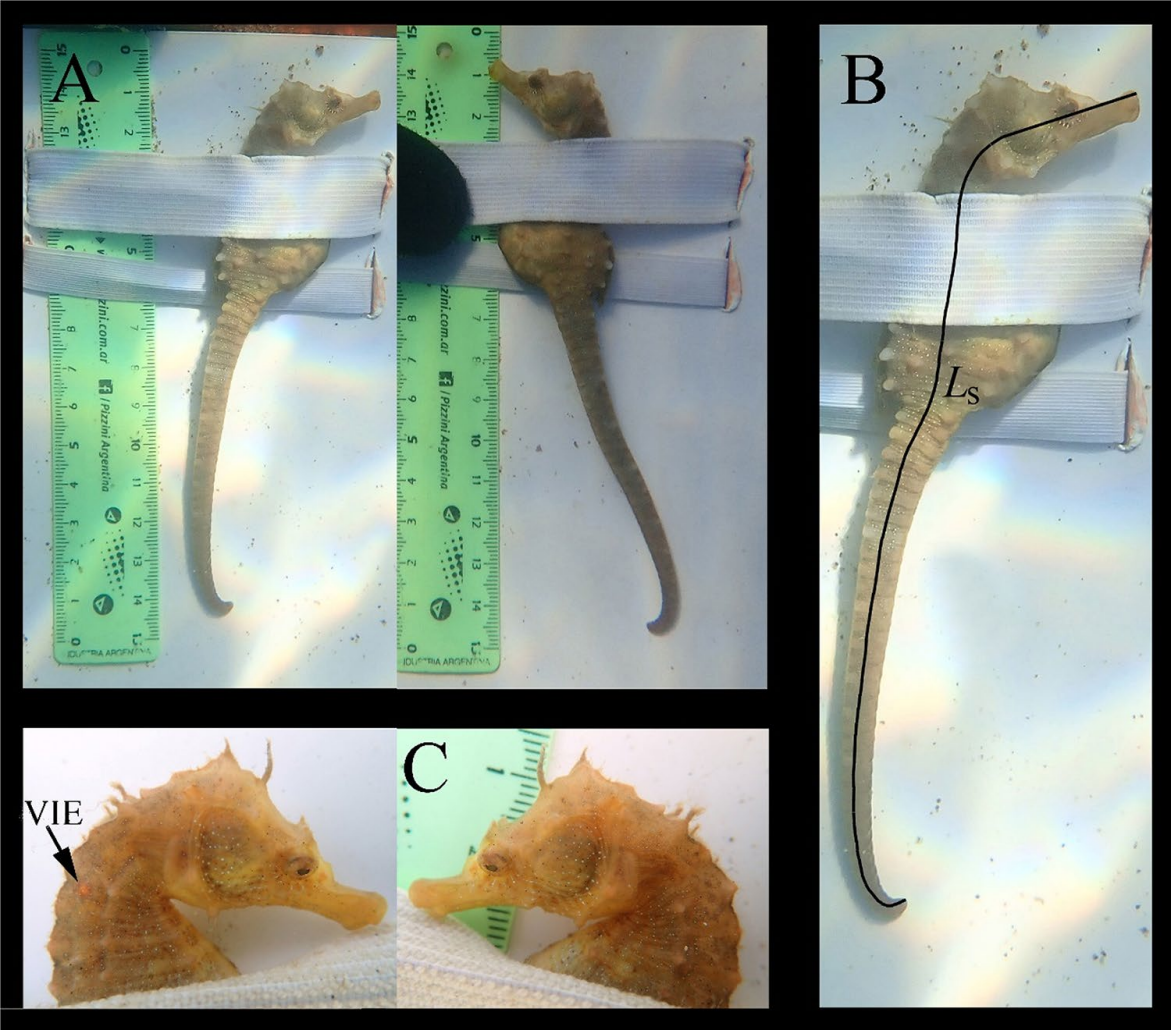
Methodological photograph of each sighted seahorse indicating: (**A**) The complete right and left seahorse profiles against the scale. (**B**) The single continuous line used in Image J for measuring the *L*_s_ of each photographed seahorse. (**C**) The right head profile showing a red visible implant elastomer (VIE) tag used in 100 individuals to validate the ability of the white dot pattern to identify individuals^10^.

Every sighted seahorse was captured by hand and gently placed on a scaled measuring board. It was secured laterally to the measuring board using a 2 cm width elastic band (fixed to the board) that was carefully wrapped around its trunk. Several lateral photographs of the entire seahorses were taken using an Olympus TG-5 camera, capturing them against the scale, as well as macro photographs of their heads from both the right and left sides (Fig. 1A). After the photographs were taken, the seahorses were immediately released, and a final photograph was captured to record their escape behavior (Supplementary Video S1). Additionally, between October 12, 2018, and December 22, 2018, a total of one hundred seahorses were uniquely tagged with red and green VIE tags.

### Methodological statements

The Institutional Review Board at CONICET (DI-2022-1580-APN-GRH#CONICET resolution) approved the work plan under the methodology proposed in this study. Fish were captured (and released) following institutional guidelines for animal welfare and regulations outlined in Argentine National Law No. 14,346. Additionally, data collection was conducted in adherence to the current regulations of the Natural Protected Area of Bahía San Antonio^20^. All animal manipulations were in compliance with the U.K. Animals (Scientific Procedures) Act, 1986, EU Directive 2010/63/EU for animal experiments, and the National Institutes of Health guide for the care and use of laboratory animals (NIH Publications No. 8023, revised 1978) guidelines. The methods reported herein also align with ARRIVE guidelines.

### Data analysis

Following each field survey, the captured photographs were promptly downloaded. The best photographs of the complete left and right body profiles, as well as the left and right head profiles, for each sighted seahorse were selected for analysis and compiled by date in a repository of all the photographs used^21^.

The time of each photograph was obtained from the metadata file. Each survey began and ended with a photograph to determine the total duration. The “individual sampling time” was calculated as the time elapsed between the first and last photograph taken for each captured seahorse, while the “searching time” was the sum of all the time periods excluding the “individual sampling times.” Additionally, the metadata from the photographs included the environmental temperature.

Standard length (*L*_s_) measurements of seahorses were conducted using Image J software. From the left and right profile photographs, the *L*_s_ was measured as a continuous line, including the head length (*L*_h_: tip of the snout to the operculum) and then along the body axis to the end of the tail (Fig. 1B). A pairwise t test was applied to compare the measurements between the two profiles. An unpaired t test was used to compare the average *L*_s_ between males (with brood pouch present) and females (with brood pouch absent).

The right head profile photograph of each sighted seahorse was printed, numbered, and organized by date to facilitate comparisons with future sampled seahorses, allowing for the identification of recaptures based on the pattern of white bright dots present on their heads^9^ (Fig. 1C). Additionally, the head contour, as well as the position and shape of spines and coronet, were compared. In cases where a recapture was identified, the left head profile photographs served as a double check. When a VIE tagged individual was recaptured, the correspondence between the tag and the pattern of bright spots on the head was verified.

Individual growth (mm) was calculated as the difference between the recaptured *L*_s_ and the initially sighted *L*_s_. The growth rate (mm/day) was determined by dividing the individual growth by the number of days elapsed between the first sighting of the seahorse and its recapture. The specific growth rate (day^−1^) was calculated by dividing the growth rate by *L*_s_.

ANOVA and paired t test were used in order to compare means. Linear regression were used to analyse the relationships with *L*_s_ and temperature. In cases where the assumptions of normality and homoscedasticity failed, the Mann–Whitney Rank Sum Test and Spearman Rank Order Correlation were applied. Variable results are reported as mean ± standard deviation. Note that the references to seasons are for the southern hemisphere.

## Results

### Sampling

During the study, 152 sampling events were conducted, resulting in a total of 976 seahorses being sighted. Among them, 488 (50%) were females and 477 (49%) were males, while the remaining 11 individuals were the smallest juveniles for which it was difficult to determine their gender based on the presence or absence of the brood pouch.

The average total time per field survey was 121 ± 30 min, and the total time expended per sampled seahorse was 1.3 ± 0.4 min (while attached to the measuring board). On average, 7.2 ± 4.6 seahorses were sampled in each field survey, with approximately 93% of the time was spent actively searching for seahorses.

**Figure 2:**
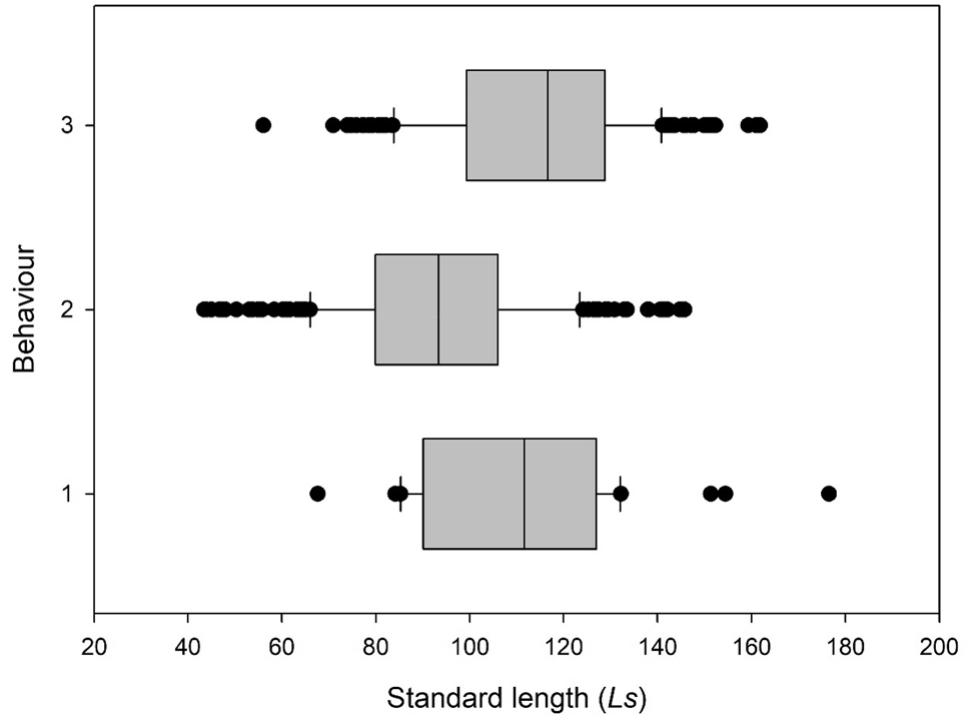
Seahorse behaviors after release from the scaled measuring board: 1. Remaining rigid on the bottom and then grabbing a nearby holdfast. 2. Swimming slowly to a nearby holdfast. 3. Escaping far away. Seahorse *L*_s_ corresponding to the observed behaviors. Median, quartiles, and data outside the 5th and 95th percentiles are indicated.

Three distinct behaviors were observed upon the release of seahorses from the scaled measuring board: escaping far away (42.4%), swimming slowly up to a nearby holdfast (49.1%), or staying rigid on the bottom for a period of time and then grabbing a nearby holdfast (8.6%) (Supplementary Video S1). A significant difference was observed in the *L*_s_ of seahorses displaying these three behaviors (one-way ANOVA; N = 479; F_2, 476_ = 55.883; *P* < 0.001), with the *L*_s_ of seahorses displaying the “swiming slowly up to a nearby holdfast” behavior being smaller than those corresponding to the other two behaviors (All Pairwise Multiple Comparison Procedures; Holm Sidak method; *P* < 0.05, Fig. 2).

### Measurements

No significant difference was found between the *L*_s_ of the left and right seahorse (Paired t test, t = 0.94, *P* = 0.17, N = 290), with a mean difference of 0.5 ± 1.1 mm (95% confidence interval). Males were larger than females (Unpaired t test, *P* = 0.006; N = 948), with a mean *L*_s_ of 102 ± 23 mm for females and 106 ± 23 mm for males.

### Recaptures

**Figure 3:**
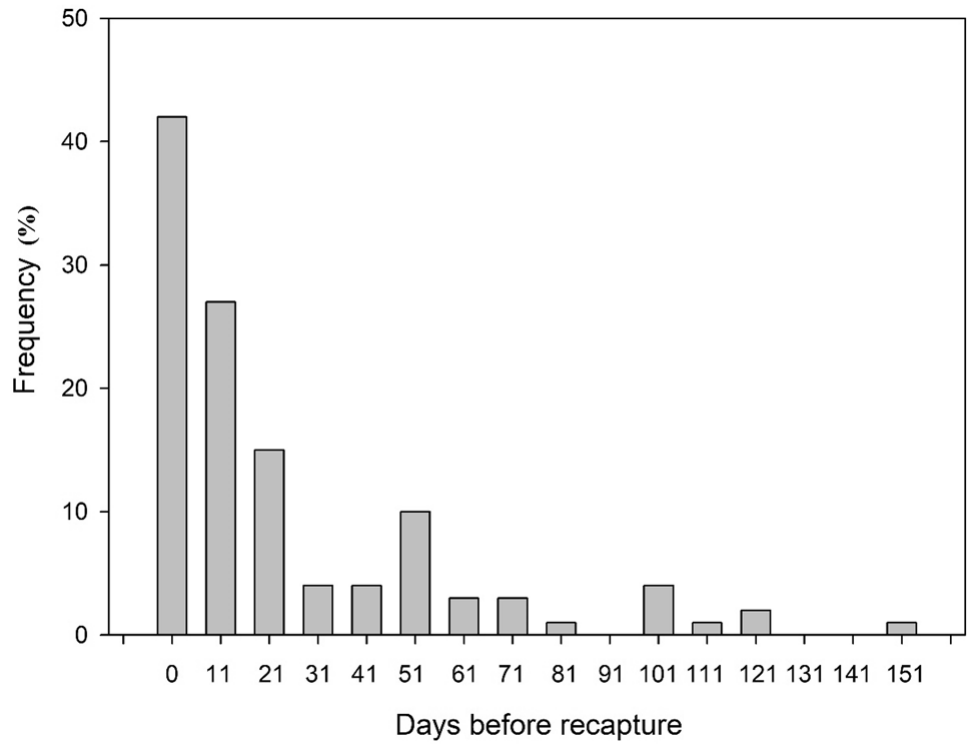
Frequency of re-sighted seahorses from the original first sighting.

The overall recapture rate was 12%, with 89 individuals being sighted more than once. Out of these recaptures, 21 were recaptured twice, five were recaptured three times, and one was recaptured four times. Thirty-nine of these recaptures were seahorses tagged with VIE tags, but two of them had lost their tags and were recognized by their head spot patterns. The remaining 37 VIE tagged seahorses that were recaptured were recognized by both methods, except for a single seahorse in which the head photographs of the recapture were dark and out of focus, making the white dot pattern indistinguishable. Most of the recaptures occurred within the first 50 days after their initial sighting, but there were some recaptures that extended beyond 100 days (Fig. 3), with one case even reaching a year.

### Growth

**Figure 4:**
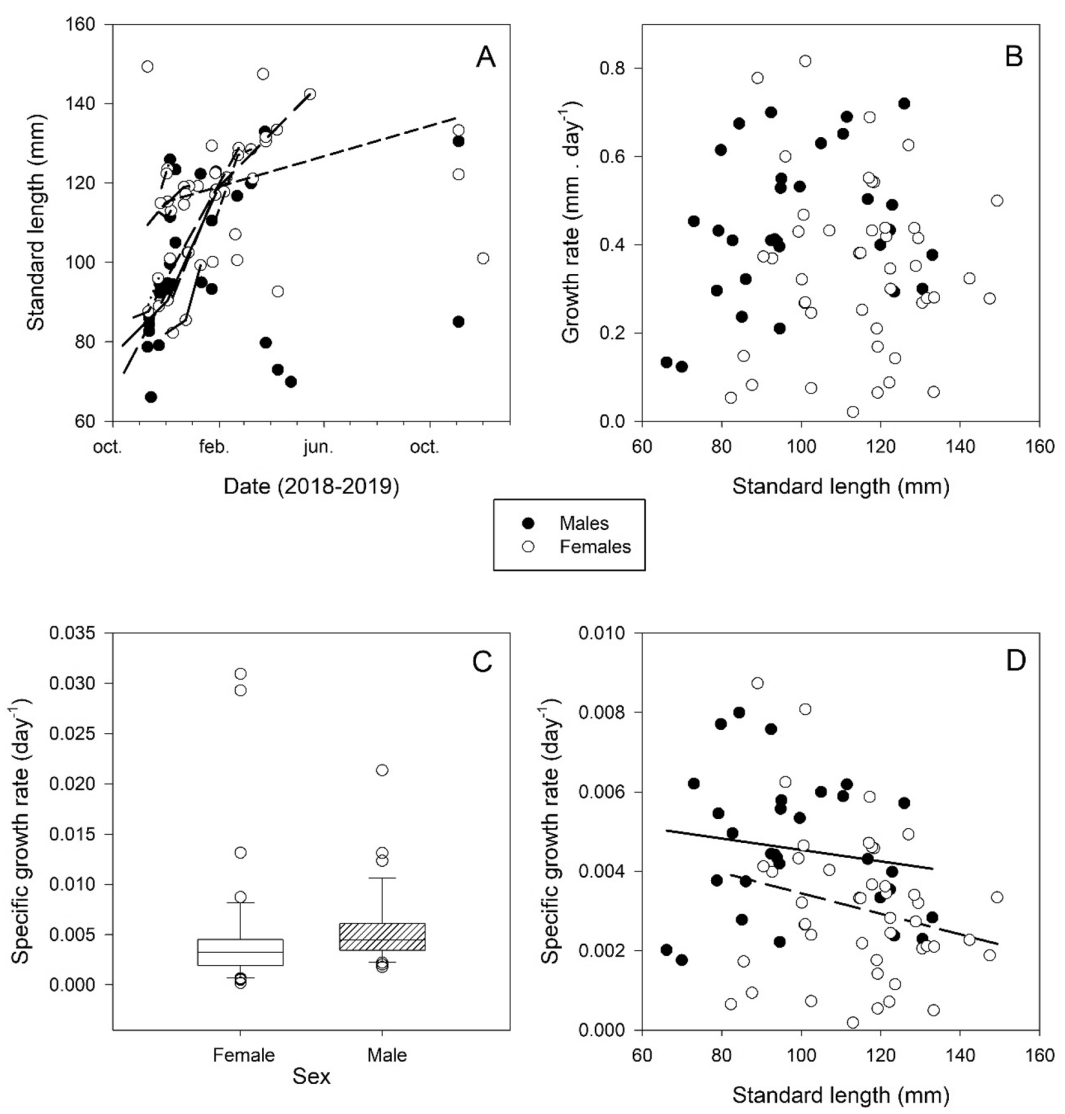
Growth of *Hippocampus patagonicus* (male: black circles; female: white circles). (**A)** Standard length (*L*_s_) versus date of male and female recaptured individuals. Lines indicate the sizes of individuals recaptured more than once. (**B**) Size specific growth rate (mm/day) versus *L*_s_ (mm) of recaptured individuals. (**C**) Specific growth rate (day-1) of male and female recaptured individuals. Median, quartiles, and data outside the 5th and 95th percentiles are indicated. (**D**) Specific growth rate (day-1) versus *L*_s_ (mm) of male and female recaptured individuals. Fitted lines are shown to indicate the trend.

Repeated recaptures allowed for the identification of two different growth patterns within the same reproductive season. The first pattern (spring, summer) exhibited fast growth, while the second pattern (late summer) showed significant size increase only after winter (Fig. 4A). The growth rate did not exhibit dependence on *L*_s_ (Regression, N = 75; F = 0.0189; *P* = 0.891; Fig. 4B). The specific growth rate was higher in males than in females (Mann–Whitney Rank Sum Test, T = 1689.000; N(male) = 33; N(female) = 48; *P* = 0.001; Fig. 4C) and was negatively correlated with *L*_s_ (Spearman Rank Order Correlation Coefficient = − 0.356; N = 76; *P* = 0.00166; Fig. 4D). No significant correlation was observed between specific growth rate and water temperature (Regression, N = 76; F_1, 74_ = 0.160; *P* = 0.691).

## Discussion

Photography has gained relevance in the study of live fishes, including morphometrics and taxonomy^22^. The profiles and head in situ photographs used in the present report allowed for quick and objective ex situ measurements, which were used to calculate the growth of an individual upon recapture. This measuring method also detected a small difference in *L*_s_ between sexes.

There have been several precedents for measuring the *L*_s_ of seahorses, as analysed here:

1. Holding the seahorses against a slate and used calipers for in situ measurements while the animals were stretched^7^. Unfortunately, at that time, the technology for current underwater compact cameras had not yet been developed, resulting in low and subjective accuracy of the in situ measurement method. Although the described in that report/review did not lead to the accurate measurement of seahorses, the observed stretching behavior when pressing the individual against a flat surface, without mentioning any specific species, could imply that this methodology might be widely applied within this fish genus.
2. Photographs of seahorses near a ruler, placing the animals inside a semi-rigid plastic bag for subsequent measurements using morphometric analysis software^23^ (tpsDig2 1.11). While this methodology seems similar to the one applied in the present report, there was not a comprehensive description of it, and its reported use was limited to distinguishing juveniles from adults. Additionally, putting a seahorse inside a plastic bag for measurement may result in increased manipulation and disturbance experienced by the animals.
3. Removing the seahorses from the water, anesthetized them for VIE tagging, and took ex-situ photographs to measure their *L*_s_ using Image J^24^. The measurement accuracy of *L*_s_ using this method would be similar to that reported in the present work. However, the method required the animals to be removed from their environment and subjected to non-measurable manipulation.

As proposed for *Hippocampus guttulatus*, the distinct bright spot pattern on the heads served as a fingerprint that persisted over time and allowed for individual identification upon recapture^9^. The visual comparison indicated correspondence between the VIE method and the unique spot pattern in recaptured individuals, demonstrating that the applied methodology was valid and, as stated above, minimally invasive. Furthermore, the identification and *L*_s_ measurement results were derived from the analysis of consecutively taken photographs, resulting in minimal seahorse manipulation time, which could enhance survey efficiency.

The impact of manipulation on an individual, as observed through their behavior immediately after release from the measuring board, showed that small individuals tended to stay near the release point, while large seahorses tended to escape. These behavioral differences between large and small individuals could be related to different age-related responses. Small seahorses may try to remain cryptic in the presence of a disturbance, while large seahorses tend to flee from it. Although speculative, this behavior could be related to different age-related responses to potential predators. It is worth noting that there has been only one recorded instance of predation on this seahorse species, where a small shark (*Mustellus schmitti*) had in its stomach content two small seahorses^25^.

This measuring and identification method would be applied in conjunction with a quantitative approach for estimating abundance and other ecological parameters^26^ to gain a more comprehensive understanding of the life history traits of seahorse populations. However, it is important to note that the method elicited different behavioral responses after manipulation and may have an impact on the original individual's position, such as “escaping far away”, which could potentially affect home range estimations.

The characteristics of this technique, which include being harmless since it removes the need for VIE tagging or any other tagging technique, being time effective, and easy to apply with minimal training, make it potentially suitable for broad use. The construction of image repositories for other populations or species through a citizen science program could be implemented, for example, by engaging dive organizations.

### Supplementary Information

Supplementary Video 1.

Supplementary Legends.

## Data Availability

The datasets used and/or analysed during the current study available from the corresponding author on reasonable request. Additionally, all the right profiles and heads photographs were lodge in a public repository^20^.
