# Supplementary material for: A novel non-invasive efficient photography-based technique for length measuring and individual identification of seahorses
Source: Sci Rep. 2023 Oct 21;13:18017. doi: 10.1038/s41598-023-45420-9 (PMC10590381; doi:10.1038/s41598-023-45420-9)
Supplement: Supplementary file 2 — Supplementary Legends. [file 41598_2023_45420_MOESM2_ESM.docx]

**Supplementary Information**

Supplementary Video S1: Video showing the photograph sampling procedure following the three possible seahorse behaviors after release. The order shown in the video is: “swimming slowly up to a nearby holdfast” followed by “escaping far away” finalizing with “staying rigid on the bottom for a period of time”.
